# Pancreaticoduodenectomy with preoperative total embolization of the hepatic arteries (PD-HAE)—a novel treatment with sacrifice of the hepatic arterial blood supply without the need for arterial reconstruction

**DOI:** 10.1007/s00423-023-03054-5

**Published:** 2023-08-15

**Authors:** JH Storkholm, SK Burgdorf, PN Larsen, CP Hansen

**Affiliations:** 1 Department of Gastroenterological Surgery and Transplantation CTx, Rigshospitalet, Copenhagen, Denmark; 2 Department of HPB Surgery, Imperial College, Hammersmith Hospital, London, UK

**Keywords:** Pancreas, Cancer, Surgery, Arterial resection

## Abstract

**Abstract:**

**Purpose:**

Tumors with involvement of common hepatic and gastroduodenal arteries (CHA and GDA) or GDA and the proper hepatic artery (PHA) are traditionally considered nonresectable. We have devised a new procedure that includes pancreaticoduodenectomy with preoperative hepatic artery embolization (PD-HAE) to facilitate an R0 resection of tumors involving the hepatic arteries without vascular anastomoses and complete sacrifice of normal hepatic arterial blood supply.

**Methods:**

To allow resection of the hepatic arteries, preoperative embolization of the PHA was performed to induce an increased collateral arterial blood flow from the periphery of the liver, far from the hepatic hilum 10–14 days prior to the operation. Between May 1, 2017 and December 31, 2019, eight patients with ductal adenocarcinoma were operated with the PD-HAE procedure.

**Results:**

The embolizations were uneventful apart from a transient marginal elevation of alanine aminotransferase in three patients.

All patients had N disease with perineural invasion of tumor cells around the adventitia of the artery and severe perivascular inflammation.

An R0 resection (> 1.0 mm to all resection margins) was obtained in six patients (75%). Mean hospital stay was 12 days. Median survival was 23 months (95% CI: 19.5–26.5 months).

Six patients (75%) are still alive 11 to 36 months after the operation. There was perioperative fatality, and morbidity was comparable to standard pancreaticoduodenectomy.

**Conclusion:**

PD-HAE is a safe procedure and may provide the opportunity for curative resection in otherwise unresectable patients. However, larger studies are needed to evaluate this procedure.

## Introduction

Despite all the advances in modern medicine, pancreatic ductal adenocarcinoma (PDAC) continues to have a poor prognosis, and the incidence is on the rise. In 2020, pancreatic cancer accounted for 2.6% new cancer cases and 4.7% of cancer deaths worldwide, making it the seventh leading cause of cancer-related deaths [1]. Although diagnosis and treatment have improved over the past years, the overall 5-year survival remains roughly 10% [2]. The main difficulty in the treatment of PDAC lies in the late detection of most cases, due to the unspecific symptoms especially tumors located in the body and tail of the gland, the early local spreading through perineural and venous invasion, and its rapid (metastatic) progression [3–5]. The generally poor response to oncologic treatment marks another obstacle, making surgery the only potential way to treat pancreatic cancer in a curative manner. At the time of diagnosis, only about 20% of patients have a surgically resectable disease [4, 6]. Along with neoadjuvant regimens, the resection of encased visceral arteries in patients with borderline or locally advanced disease is a new approach to treat otherwise palliative patients.

Resection of the peripheral branches of the celiac axis (CA) is performed routinely in cases with tumor invasion of the splenic artery [7]. However, invasion of the common hepatic artery (CHA) often presents a more complex problem. Locally advanced cancer in the body of the pancreas commonly involves CHA and/or CA often with perineural invasion of the central nerve plexus that surrounds the arteries. These patients may be eligible for distal pancreatectomy with celiac axis resection (DP-CAR) [7–9], provided that the gastroduodenal artery (GDA) is without invasion, as liver flow can be maintained via the inferior pancreaticoduodenal arcades arising from the superior mesenteric artery (SMA) connecting the GDA to the proper hepatic artery (PHA). However, DP-CAR is usually not possible in patients with involvement of CHA/GDA/PHA zone.

From experience with palliative treatment of hepatocellular carcinomas, spontaneous arterial revascularization of the liver occurs after ligation of the PHA. Pre-operative embolization of the PHA seems to promote increased arterial collateral blood flow in preexisting collaterals transversing the hepatic suspensory ligaments, the retroperitoneum, and the hepatoduodenal ligament, taking over the arterial supply of the liver [10–14].

We have devised a new procedure to facilitate R0 resection of pancreatic tumors involving the GDA, PHA, and CHA. These arteries along with the left and right hepatic artery (LHA, RHA) can be completely resected without any vascular anastomoses. To allow resection of the hepatic arteries in these patients, preoperative embolization of the PHA was performed 8–10 days prior to the operation to induce arterial collateral blood flow from the periphery of the liver, far from the hepatic hilum and the tumor.

## Material and methods

Our institution is a tertiary center for hepato-biliary-pancreatic surgery affiliated with Copenhagen University and has a catchment area of around 2.8 million people.

Between January 1, 2017 and December 31, 2019, 696 patients with pancreatic tumors were operated at our institution of whom 592 patients (85.0%) had resectable disease. Eight patients had PD-HAE procedures (1%). In the same period, 104 patients (15.0%) had unresectable disease and underwent surgical exploration only.

Resectability was evaluated preoperatively at our multidisciplinary team (MDT) conference in the presence of radiologists, interventional radiologists, nuclear physicians, oncologists, and surgeons. Diagnostic imaging included a three-phase multi-detector-row computed tomography (MDCT) and, if needed, supplementary magnetic resonance imaging (MRI) and/or positron emission tomography-computed tomography (PET-CT). After the MDT conference, the surgeon and an anesthesiologist evaluated the operability of patients with resectable tumors. Preoperative risk assessment was graded according to the American Society of Anesthesiologists classification (ASA).

Operation time, perioperative blood loss, postoperative hospital stays, and complications were recorded. Postoperative complication score was based on the Clavien-Dindo classification of surgical complications [15]. The International Study Group on Pancreatic Surgery (ISGPS) classification was used to score postoperative pancreatic fistula, delayed gastric emptying, and post-pancreatectomy hemorrhage [16–18]. Surgical site infection was defined according to the Centers for Disease Control and Prevention (CDC) definitions [19]. Ischemic morbidity was defined as an abdominal organ complication caused by surgery-related ischemia. Perioperative death was defined as all deaths occurring during hospitalization and/or within 90 days of surgery. Events related to survival were measured from the time of surgery. Length of hospital stay was defined as the time from admission to discharge. Tumor stage was classified according to the AJCC cancer manual 8th edition [20]. The resection margin status (R-status) was evaluated according to the general recommendations by the Royal College of Pathologists definition and was classified as R0 (no residual, distance margin to tumor > 1 mm), R1 (residual tumor, distance margin to tumor < 1 mm), and R2 (residual tumor, macroscopically positive margin) [21].

### Preoperative embolization

All cases were seen at the MDT, and the relevant treatment options were evaluated. When a potential PD-HAE case was presented at the MDT, we carefully examined the arterial anatomy. Patients were considered eligible for PD-HAE if the tumor involved the GDA, PHA, and CHA junction and or the LHA/RHA but did not extend into the hilar plate. In the case of pre-existing-replaced RHA arising from the superior mesenteric artery (SMA), only the LHA was embolized if involved (and provided that it did not arise from the left gastric artery (LGA)).

**Fig. 1:**
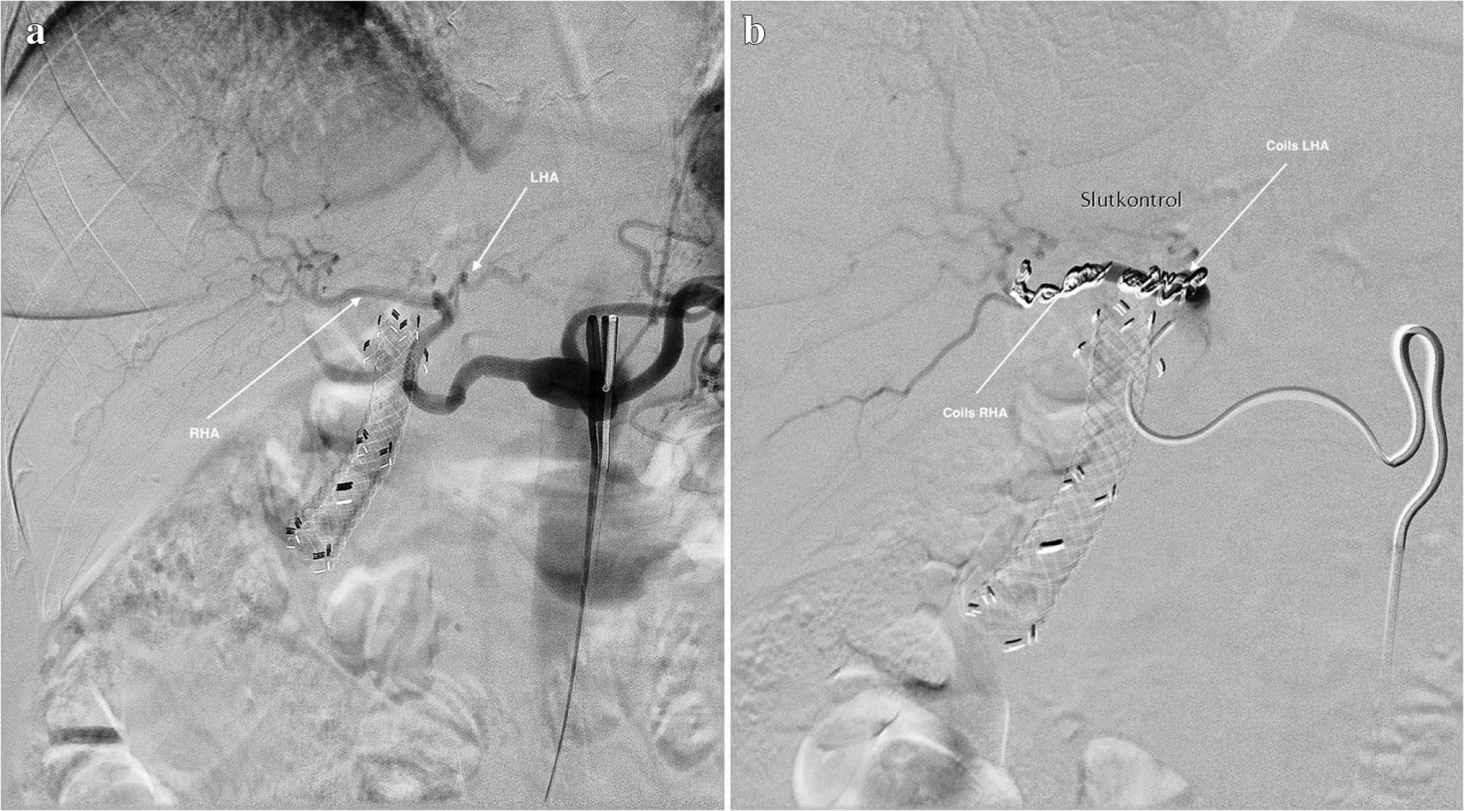
**a** Angiogram of the hepatic arteries before embolization (RHA, right hepatic artery; LHA, left hepatic artery)**. b** Angiogram after embolization (arrows depict coils in the RHA and LHA)

During this procedure, selective catheterization of PHA and RHA/LHA was performed under X-ray guidance via the femoral artery. Once at the attended level, coils were released to occlude the selected branches (Fig. 1a, b). Embolization was done around 10 days before surgery to allow enough time for collaterals to develop. Percutaneous Doppler ultrasound (DU) was conducted out the day after embolization, and liver parameters were monitored.

### Operative technique

**Fig. 2:**
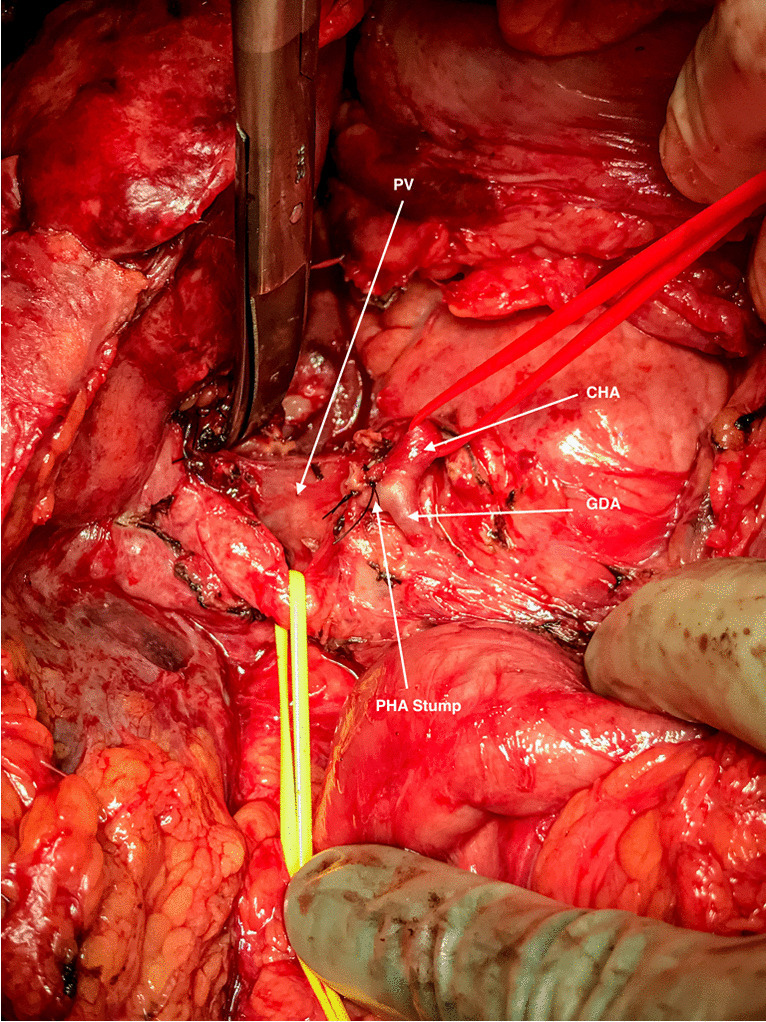
Intraoperative photo after division of the proper hepatic artery (PHA). The common bile duct has been retracted by yellow vessel loop exposing the portal vein (PV). Below the transected PHA, the gastroduodenal (GDA) and the common hepatic (CHA red vessel loop) arteries are seen

During the operation, a thorough exploration with intraoperative ultrasound was performed. All operations were performed as “artery first” procedures [22], and a wide Kocher’s maneuver and opening the lesser omentum provided access to the origin of the SMA and the CA and its branches. This allowed the dissection of the CA base to exclude tumor invasion. Biopsies were collected at the base of the hepatic hilum. If frozen sections were without tumor cells, the procedure continued. To avoid disrupting the collaterals, the liver was not mobilized nor were the hepatic ligaments divided. The superior mesenteric vein (SMV) was dissected at the lower margin of the pancreas and the portal vein dissected in the hepatoduodenal ligament. The SMA was in most cases dissected with a posterior approach. This allowed an assessment of the degree of tumor extension in the triangle between the CA, SMA, and SMV. Next, the common hepatic duct was carefully dissected, and a trial clamp was applied to the hepatic arteries while intraoperative Doppler ultrasound was performed to assure collateral hepatic arterial flow. If the collateral arterial flow was detected, the RHA and LHA were divided at the hilum (Fig. 2). All tissue in the ligament including the portal vein was, if required, resected, and an end to end anastomosis was performed above the splenic vein. The remaining steps of the procedure followed a standard Whipple procedure with pancreatico-jejunostomy, hepatico-jejunostomy, and gastro-jejunostomy.

At the time of clamping of the liver arteries, continuous infusion of Flolan® 2 ng/kg/min (epoprostenol, GlaxoSmithKline Pharma A/S, UK) was initiated. This infusion continued for 5 days. Epoprostenol is a potent pulmonary and systemic vasodilator but also reduces platelet aggregation [23, 24]. Thrombosis prophylaxis with LMW heparin, Klexane® 40 mg q.d. (enoxaparin, Sanofi S.A., FR), was started the evening before surgery and continued after infusion of Flolan® was terminated. LMW heparin was given until discharge after which further anticoagulation was not given. Postoperatively ALAT and LDH were monitored daily as was the hepatic arterial blood flow by Doppler pulsed wave ultrasound.

## Results

The study included eight patients with pancreatic ductal adenocarcinoma (PDAC). Five patients received preoperative downstaging chemotherapy four with FOLFIRINOX. Two patients also received 25 Gy external radiation, and one patient received gemcitabine and Nab Paclitaxel. Three patients did not receive preoperative chemotherapy as the tumor at the MDT was considered up-front resectable at the MDT. Six patients received adjuvant therapy (five FOLFIRINOX and one patient gemcitabine and Nab Paclitaxel).

Seven patients underwent embolization of the left and right hepatic artery separately. In the last patient, the RHA originated from the SMA and was not affected by the tumor and could be spared. Therefore, only the LHA was embolized.

In three patients, the levels of alanine aminotransferase (ALAT) were only marginally and transiently elevated after embolization and returned to normal levels in less than 24 h. Other liver parameters were unaffected. No patient had complications because of embolization of the RHA and LHA (see Table 1 and Table 2). The patients were all discharged the day after embolization.

**Table 1:** Patient characteristics, embolization type, and liver enzymes after embolization

Patient no.	Gender	Age	BMI	ASA score	Pre OP CA19-9 (U/l)	Pre OP Kemo	Pre OP bleed (ml)	OP time (mn)	Pre OP embolization	ALAT (U/l) day 0	ALAT (U/l) day 1	LDH (U/l) day 0	LDH (U/l) day 1
1	M	61	39	2	638	N	350	255	RHA + LHA	637	45	766	138
2	F	73	22	2	1987	FOLFIRINOX	1200	245	RHA + LHA	68	22	311	198
3	F	68	30	3	399	FOLFIRINOX	800	190	RHA + LHA	154	32	339	220
4	M	65	27	2	265	N	400	230	LHA only	22	27	167	138
5	M	49	29	1	6755	FOLFIRINOX + 25 Gy	1800	280	RHA + LHA	30	28	186	156
6	F	38	20	1	318	N	600	300	RHA + LHA	99	33	299	189
7	F	60	27	2	2213	Gem-Nab Paclitaxel	850	225	RHA + LHA	24	23	117	127
8	F	68	31	1	98	FOLFIRINOX + 25 Gy	700	310	RHA + LHA	29	30	232	201

**Table 2:** Delay from embolization to operation, procedure, duration of operation, bleeding, Clavien-Dindo score, pathology, and outcomes. *Thrombus in PV graft, **grade B fistula, fatal late bleeding, and ***grade B fistula; †patient had positive station 16B1 lymph node

Patient No	Days from embolization to surgery	Surgical procedure	Hospital stay (days)	Clavien-Dindo classification	AJCC stage	R status	Adjuvant chemotherapy	Recurrence	Status dead/alive
1	12	PD-HAE+PV/SMV resection w. interposition graft	12	3a*	ypT3N2M0	R1	FOLFIRINOX	Liver met 9 mo.	Alive 23 months
2	10	PD-HAE	9	0	ypT3N1M0	R0	FOLFIRINOX	No	Alive 16 months
3	10	PD-HAE	8	0	ypT3N2M0	R0	FOLFIRINOX	Local recurrence 10 mo.	Alive 28 months
4	14	PD-HAE	13	5**	ypT3N2M0	R0	No	-	Dead POD 18
5	9	PD-HAE	11	0	ypT4N2M1*	R0	Gem+Nab Paclitaxel	No	Alive 23 months
6	10	PD-HAE	11	0	ypT3N2M0	R0	FOLFIRINOX	-	Dead 36 months
7	8	PD-HAE+PV/SMV resection	14	0	ypT4N2M0	R0	No	No	Alive 20 months
8	10	PD-HAE+PV/SMV resection	18	1***	ypT4N1M0	R1	FOLFIRINOX	No	Alive 11 months

Postoperatively, ALAT and LDH levels were completely unaffected in seven patients. In one patient, there was a transient rise of ALAT and LDH peaking at 987 and 766, respectively (please see Tables 1 and 2). This corresponded with the thromboembolic episode and subsequent minor anterior infarction of the liver described below. All patients had normal bi-phasic bilateral arterial curves on pulsed wave Doppler ultrasound from POD 1 onwards.

Histological examination of the specimens showed invariably ductal adenocarcinoma.

Six patients (75%) had R0 resections, and two patients had R1 resections. No patient had tumor invasion at the posterior margins.

All patients had lymph node involvement. Six had N1 disease, and two had N2 disease. One patient with N2 disease also has a positive station 16B1 and was, therefore, classified as M1. Histological evaluation invariably showed perineural invasion and invasion of the tunica muscularis as well as severe inflammation with luminal narrowing of the offended vessel (RHA, LHA, and PHA). In three cases, this invasion extended to the CHA. These patients were, thus, classified as T4.

One patient had a 12 cm long portal interposition graft (from deceased donor) but developed a thrombosis in the graft on POD 4. This was treated with radiological interventional thrombectomy. The patient developed a minor infarction of the anterior parts of the liver but recovered. The average hospital stay was 12 days.

**Fig. 3:**
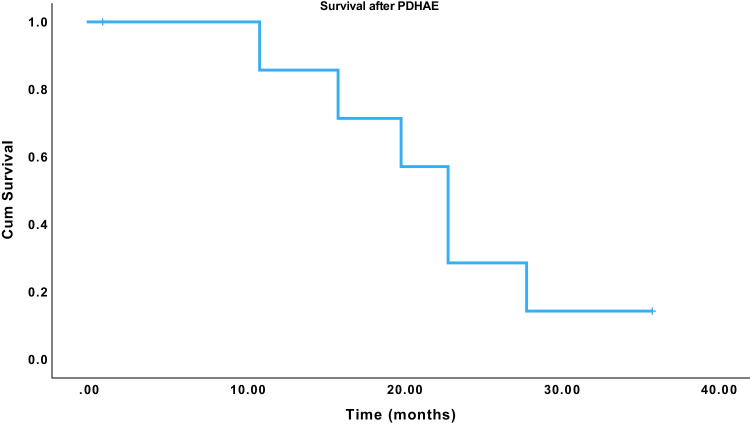
Survival plot after PDHAE. Median survival was 23 months (95% CI: 19.5–26.5 months)

There was one peri-operative death from a massive late hemorrhage on POD 18 from a pseudoaneurysm on the splenic artery. This patient also had a grade B postoperative pancreatic fistula. One patient died 35 months postoperatively from liver and lung metastases. The remaining patients are still alive. However, one patient developed liver metastases 9 months after surgery, and another patient had an isolated local recurrence 10 months after surgery. This patient was subsequently treated with radiation therapy and is still alive. Median survival was 23 months (95% CI: 19.5–26.5 months; please see Fig. 3).

## Discussion

To allow complete resection of the hepatic artery, the patients had preoperative embolization of PHA or RHA and LHA to induce arterial collateral blood flow from the periphery of the liver. Previous studies have examined the safety of ligation and embolization of the hepatic artery, the changes in hepatic hemodynamics after ligation, and the collateral pathways. Older studies have described 26 arteries that could be detected as collateral pathways after occlusion of the PHA [10–14].

From experiences with reconstruction of the hepatic arteries in pancreatic surgery, the rate of failure and low patency rate of arterial anastomoses with the added risk of liver failure in these patients are relatively high [25]. Even after successful arterial reconstruction, median survival has been poor with no long-term survivors since most cases are R1/2 resections [26, 27]. Preoperative total hepatic arterial embolization has previously been performed in surgery for hilar cholangiocarcinoma [28]. However, arterial reconstruction in pancreatic surgery is associated with an increased morbidity and mortality, which can be attributed to bleeding complications and thrombosis [29]. Because these complications imply a considerable risk of a lethal outcome if the patient develops postoperative pancreatic fistula, total pancreatectomy should be considered when complex arterial reconstruction is performed [30].

Portal vein arterialization has previously been demonstrated as a salvage procedure after disruption of arterial supply to the liver but has severe drawbacks such as prolonged over-arterialization causing hyperbilirubinemia and other side effects, if the shunt is not closed within a few weeks [31, 32]. Since the patients in this study did not have liver resections and the liver was not mobilized, we could safely rely on the collateral arterial blood supply. However, as it was demonstrated in the patient with portal vein thrombosis and subsequent minor anterior liver infarction, maintaining a sufficient portal venous flow plays a crucial role in an arterially challenged liver.

A preoperative redirection and preconditioning of the blood flow to the liver may cause only a minor reduction in organ perfusion compared to the sudden and pronounced changes that may occur in central splanchnic hemodynamics during an operation [33–35].

Indeed, we observed only one minor ischemic infarction anteriorly in the liver in one patient with portal vein graft thrombosis. The use of Flolan® as a platelet aggregation inhibitor and systemic vasodilator may have further reduced ischemic complications. This will, however, need further study.

An alternative to arterial resection with or without reconstruction is arterial divestment which employs dissection and removal of the periarterial neurolymphatic tissues off the arterial wall without any vascular resection [36]. The authors describe that a tumor-free dissection plane is opened by blunt or sharp dissection and can be followed by circumferential dissection intimately alongside the vessel wall to dissect areas of tumor vessel contact. [37] These techniques have mainly been used on the SMA, celiac trunk, or the CHA. However, in small thin-walled arteries such as RHA, LHA, and PHA with invasion of the tunica muscularis, this would certainly appear to compromise the oncological result. Moreover, the risk of injury to the vessel and subsequent development of pseudoaneurysms seems not worth the risk.

A previous study from Japan used preoperative embolization of the CHA or PHA in 21 patients with suspected arterial invasion as preconditioning before arterial resection and reconstruction during the subsequent surgical procedure [38].

Yoshidome et al. performed preoperative embolization of the CHA and resection of this vessel in a series of seven patients [39]. The study had several ischemic liver complications in patients undergoing total pancreatectomy mainly because the retroperitoneum around the coeliac axis was dissected. This may compromise the collateral arterial supply especially to the left liver.

In this series, the preoperative arterial embolization allowed aggressive dissection in the liver hilum, without the need to worry about preserving the normal arterial blood supply, to achieve the best oncological result, since the collateral arterial blood supply after embolization was far from the hepatic hilum. This may also explain the very high rate of R0 resections.

Apart from one fatal outcome due to late bleeding from a ruptured splenic aneurism, our patients had morbidity comparable to standard Whipple procedures. One patient had a localized necrosis in the anterior aspects of the liver but recovered without further intervention.

When the hepatic arteries are embolized, the liver will rely on collateral arterial blood supply. The main portion of this supply comes via the hepatic ligaments, the retroperitoneum, but also via the hepatoduodenal ligament (HL). The main collaterals from the HL are the small arteries surrounding the CBD and CHD. The trial clamp was applied to ensure that the collateral blood supply via the non-HL routes was sufficient.

The criterion for sufficient collateral flow was a detectable bi-phasic arterial signal using pulsed wave Doppler ultrasound. Moreover, biliary arterial blood flow is normally maintained only by the hepatic arteries. We did not observe any rise in bilirubin postoperatively, and more importantly, we did not see any complications related to the hepatico-jejunostomy implying a sufficient retrograde biliary arterial blood supply via the collateral arterial system.

We achieved a high proportion of R0 resections (75%) in these advanced tumors. The main reason for this high R0 rate may be that the hepatoduodenal ligament could be dissected very aggressively or resected completely. This dissection could be extended into the hilar plate with venous and bile duct reconstruction only. Furthermore, PD-HAE does not involve a risky arterial anastomosis which may necessitate a total pancreatectomy.

The levels of complications compared well to a Japanese study with preoperative embolization of the CHA (39). In this study, median survival was 12.6 months, and our median survival was 23 months. However, any definite conclusion cannot be drawn from this comparison since the number of patients in both cohorts was very low (7 vs. 8 patients).

## Conclusion

PD-HAE seems safe and is a procedure which may be useful in patients with otherwise unresectable pancreatic tumors at the upper margin of the pancreatic head that involve the CHA/GDA/PHA and where resection of both hepatic arteries is necessary to achieve an R0 resection. However, this is a very limited series, and larger prospective studies are needed to further evaluate this procedure.
